# Effects of light intensity on the growth of *Polygala fallax* Hemsl. (Polygalaceae)

**DOI:** 10.3389/fpls.2022.985628

**Published:** 2022-08-26

**Authors:** Huiling Liang, Baoyu Liu, Chao Wu, Xiujiao Zhang, Manlian Wang, Xiyang Huang, Li Wan, Hui Tang

**Affiliations:** Guangxi Key Laboratory of Functional Phytochemicals Research and Utilization, Guangxi Institute of Botany, Guangxi Zhuang Autonomous Region and Chinese Academy of Sciences, Guilin, China

**Keywords:** *Polygala fallax* Hemsl., light intensity, photosynthesis, biomass, survival rate, saponin

## Abstract

*Polygala fallax* Hemsl. (Polygalaceae), a traditional Chinese medicinal species, requires optimal growth conditions for artificial cultivation. Irradiance is one of the primary environmental factors that affects the growth and survival of *P. fallax* Hemsl. plants, which seemingly grow better under weak irradiance conditions. However, the optimum light intensity for growing *P. fallax* Hemsl. is not clear. To determine the optimum light intensity for cultivating this medicinal plant species, *P. fallax* Hemsl. plants from two different habitats were grown and exposed to three shade treatments (50% shade, 70% shade and 90% shade, which resulted in photosynthetically active radiation amounts equal to 662 μmol m^−2^ s^−1^, 401 μmol m^−2^ s^−1^, and 131 μmol m^−2^ s^−1^, respectively) to evaluate survival, growth, leaf photosynthesis, and the main pharmacological active ingredients (saponins) in response to shade. Our results revealed that the *P. fallax* Hemsl. plants in the different habitats consistently exhibited relatively high photosynthesis rates, biomass, survival rates and saponins under 662 μmol m^−2^ s^−1^ created by the 50% shade treatment. We concluded that photosynthetically active radiation of approximately 662 μmol m^−2^ s^−1^ is suitable for the cultivation of *P. fallax* Hemsl. plants.

## Introduction

*Polygala fallax* Hemsl., a species of the Polygalaceae family, is a shrub or small tree distributed mainly in southern China (Xu et al., 2003; Zhang et al., 2012). In traditional Chinese medicine, the roots of *P. fallax* Hemsl. have been used in the treatment of acute and chronic hepatitis (Zhang et al., 1997; Lin et al., 2005), as they have certain pharmacological effects, such as anti-HBV, immune regulation and antioxidant activities (Hong et al., 2014; Yao et al., 2020). Pharmaceutical analysis shows that there are several natural active ingredients, particularly saponins, which have great pharmacological activities, in *P. fallax* Hemsl. plants (Ma et al., 2003; Podolak et al., 2010). Additionally, *P. fallax* Hemsl. is edible and has been used for both medicine and food in the local folklore of Guangxi. Thus, *P. fallax* Hemsl. has great medicinal and economic value, and there is great utilization potential that warrants further exploration for improving global food security and public health (Fahad et al., 2021).

In recent years, the amount of wild resources of *P. fallax* Hemsl. has sharply declined due to overexcavation. Thus, there is an urgent need to cultivate *P. fallax* Hemsl. plants to meet the increasing demand and protect wild resources. However, low survival rates were frequently observed when the wild *P. fallax* Hemsl. plants were transplanted to smallholder farms, and the biomass yield of the surviving plants was relatively unstable and generally low under natural field conditions (Wang et al., 2016). Zhang (2013) reported that wild *P. fallax* Hemsl. populations were found to be mainly distributed in the shaded and humid environments of valley forests. As such, the growth of *P. fallax* Hemsl. plants requires relatively harsh environmental conditions.

Recently, *P. fallax* Hemsl. plants were reported to grow better under weak light conditions than under high light conditions (Wang et al., 2016). We thus speculated that irradiance is one of the primary environmental factors that affects the survival and growth of *P. fallax* Hemsl. plants. However, the optimum light intensity for the growth of *P. fallax* Hemsl plants is not clear. Light is crucial not only for leaf photosynthesis, plant growth, and biomass accumulation but also for the production of secondary metabolites in medicinal plants (Fahad et al., 2013; Müller et al., 2013; Townsend et al., 2018). However, few studies have evaluated the effects of light intensity on the growth and active ingredients of this traditional Chinese medicinal plant species (Zhang, 2013; Wang et al., 2016). The objectives of the present study were to (i) evaluate the effects of different light intensities on plant growth and the active ingredients in plant tissues of *P. fallax* Hemsl. plants and (ii) determine the optimum light intensity for the production of *P. fallax* Hemsl. plants.

## Materials and methods

### Plant material and growth conditions

Pot experiments were conducted at Guangxi Institute of Botany, Chinese Academy of Sciences, Guilin, China (25°4′N, 110°18′E, 175 m above sea level) from 2019 to 2021. The materials included two *P. fallax* Hemsl. plants collected from two locations, i.e., Lingchuan (LC) county in Guiling city (25°25′N, 110°20′E) and Zhaoping (ZP) county in Hezhou city(24°11′N, 110°48′E), which are the original habitats for wild *P. fallax* Hemsl. plants in Guangxi Province, China. The seeds of *P. fallax* Hemsl. collected from the two habitats (LC, ZP) were sown in nursery beds at the Guangxi Institute of Botany on 16 Sept. 2019. Individual seedlings were transplanted to cylindrical pots (height, 12 cm; diameter, 8 cm) filled with 1.2 kg of clay soil on 10 March 2020 and grown under natural ambient conditions. The soil of the experimental field was a clay loam with a pH of 6.23, a total nitrogen content of 1.15 mg kg^−1^, an available potassium content of 117 mg kg^−1^, and an available phosphorus content of 11.45 mg kg^−1^.

### Shade treatment

After seedling recovery, 150 pots of seedlings with a uniform plant architecture for each *P. fallax* Hemsl. plant from each habitat were randomly arranged into three groups, with fifty pots in each group, and subjected to one of three different shade treatments, i.e., 50% shade treatment, 70% shade treatment, and 90% shade treatment, on 11 Apr. 2020. A shade net (deep colored nylon mesh) was used to reduce the full light amount by 50, 70, and 90%, which provided 50, 30 and 10% of the original light intensity, respectively. The photosynthetically active radiation (PAR) under full light averaged 1,339 ± 71 μmol m^−2^ s^−1^ (*n* = 10, the light input peaked at 1806 μmol m^−2^ s^−1^ at midday), and it was 662 ± 47 μmol m^−2^ s^−1^, 401 ± 34 μmol m^−2^ s^−1^, and 131 ± 12 μmol m^−2^ s^−1^ after the 50, 70, and 90% shade treatments, respectively. The plants were grown under the shade treatments for 6 months, after which the plants were sampled (five replications each).

### Leaf photosynthesis

Measurements of the diurnal net photosynthetic rate were performed on the youngest fully expanded mature leaves on the same side of the canopy using a portable photosynthesis system (LI-COR 6400, Lincoln, NE, United States) equipped with light and CO_2_ control modules. The block temperature and CO_2_ concentration in the cuvette were set to those of ambient conditions. The flow rate of the air was set to 500 μmol s^−1^. To prevent the influence of external interference, the air inlet of the LI-6400 was connected to a plastic tube (2 ~ 3 m in length), and the end of the tube was placed away from the operator. The artificial photosynthetic photon flux density (PPFD) was set to 0, 20, 50, 100, 150, 200, 400, 600, 800, 1,000, 1,200, 1,500, 1800, and 2000 μmol m^−2^ s^−1^ in sequence to generate photosynthetic light response curves. To promote stomatal opening, sampled leaves were exposed to natural light conditions for 20 min before measurements.

### Chlorophyll content

The fully expanded mature leaves of five plants randomly selected from each shade treatment were used to determine the chlorophyll content. Leaf discs were collected, ground to a fine powder and extracted with 10 ml of 80% acetone (v/v). The homogenate was then centrifuged at 4,000× *g* at 4°C for 10 min, after which the supernatant was separated and used for the chlorophyll assay. The amounts of chlorophyll a, b and carotenoids were determined spectrophotometrically by reading the absorbance at 663 (*W*_663_) nm, 645 (*W*_645_) nm and 480 (*W*_470_), respectively (Lichtenthaler and Wellburn, 1983). The chlorophyll content was expressed as milligrams per unit leaf area (mg dm^−2^). The calculations were as follows:


$$\mathrm{Chlorophyll}\;a\;\left(C_{a}\right)=12.21\times W_{663}-2.81\times W_{645}$$

$$\mathrm{Chlorophyll}\;b\;\left(C_{b}\right)=20.13\times W_{645}-5.03\times W_{663}$$

$$\mathrm{Carotenoids}=\frac{1,000\times W470-3.27\times Ca-104\times Cb}{229}$$

Total chlorophyll=Ca+Cb

### Extraction and determination of saponins

The total saponins in the roots and stems were extracted and quantified using a Test Kit (QYS-239346, Qiyi biological technology (Shanghai) CO., LTD., China) according to the instructions, which is similar to the method of Yu et al. (2017) with minor differences. Briefly, the sampled roots and stems were crushed and sieved by an 80 mesh sieve after drying to a constant weight at 60°C. A total of 0.05 g of powder was weighed and extracted in methanol (1 ml) with ultrasonication for 1 h. The extract solution was centrifuged at 8,000× *g* for 10 min at 25°C, after which 0.5 ml of the supernatant was collected and freeze-dried. Next, 0.2 ml of fresh 5% (W/V) vanillin-acetic acid solution and 0.8 ml of perchloric acid were added, and the mixtures were incubated at 55°C for 20 min. Finally, 0.2 ml of the cooled mixture was collected in a dark-colored tube, and 1.0 ml ethyl acetate was added. After mixing thoroughly, the absorbance was measured by an ultraviolet spectrophotometer (Shanghai Spectrum Instruments Co., Ltd.) at 589 nm, with a blank solution as a reference. The content of total saponins was expressed in units of mg per g DW.

### Growth measurements

The *P. fallax* Hemsl. plants from the two habitats (LC, ZP) were collected from each shade treatment on 27 Oct. 2020. The height, basal diameter, crown width and length and width of the fully expanded mature leaves of each plant were measured with a ruler. The root length was measured after the whole plants were rinsed with tap water. Next, the roots, shoots, and leaves were separated to determine their dry weight (DW, g), which was used to express the biomass (g plant^−1^) of the plants.

### Statistical analysis

Analysis of variance (ANOVA) for all the variables was carried out using Statistix 8.0 (Analytical Software, Tallahassee, FL, United States). The treatment means were compared using the least significant difference (LSD) test at the *p* < 0.05 level.

## Results

### Effects of light intensity on the survival of *Polygala fallax* Hemsl.

**Figure 1:**
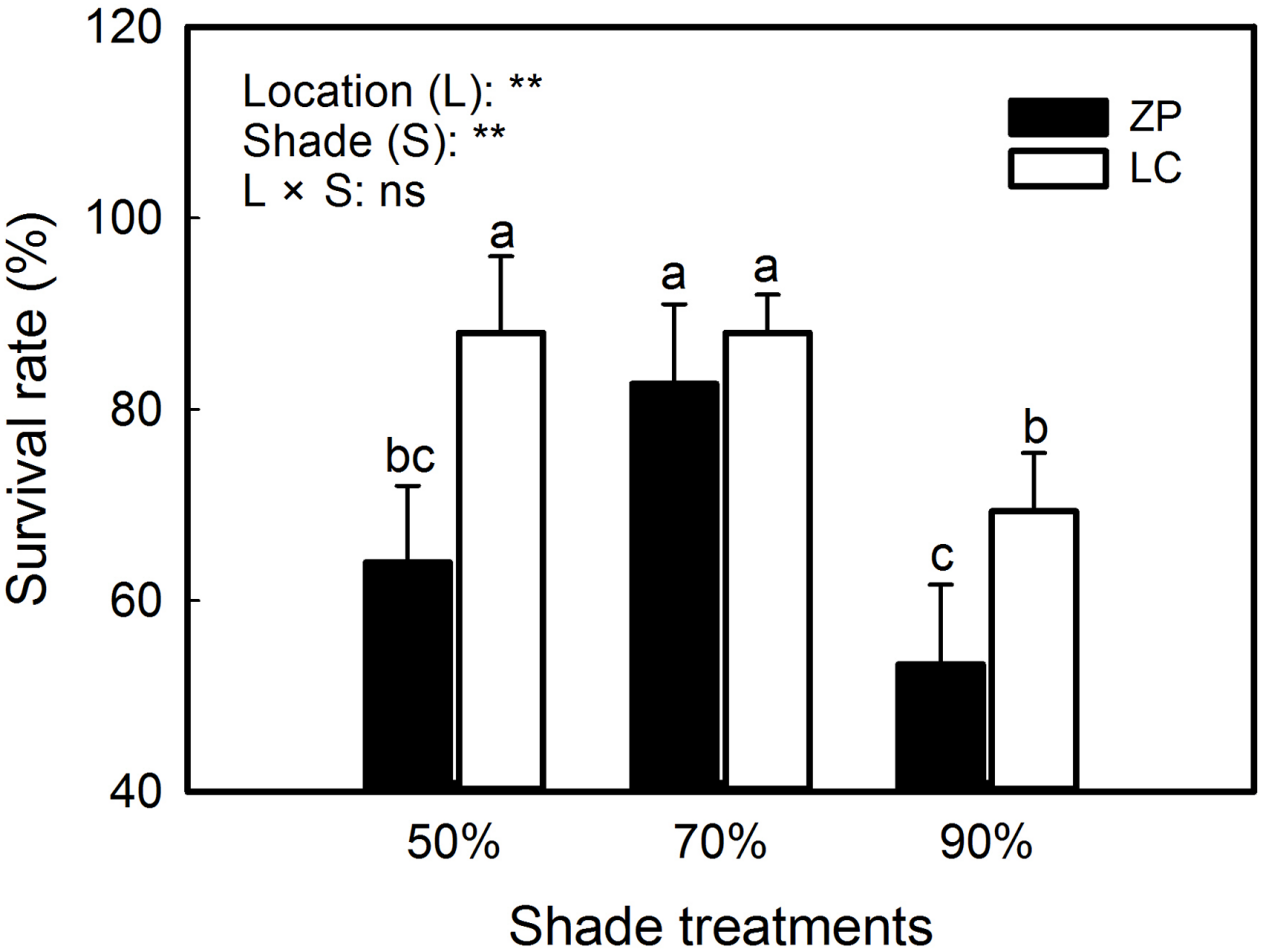
Effects of shade treatments on the survival of *Polygala fallax* Hemsl. plants. The data are presented as the mean ± SD (*n* = 5). The different letters indicate significant differences according to an LSD test at *p* < 0.05.

As shown in Figure 1, the survival rate of *P. fallax* Hemsl. plants significantly differed among the shade treatments and between the locations, and nonsignificant interactions occurred between the shade treatments and locations. The survival rates were 88.0, 88.0 and 69.3% under the 50% shade treatment, 70% shade treatment, and 90% shade treatment, respectively, in *P. fallax* Hemsl. plants from the LC habitat. However, the survival rates of *P. fallax* Hemsl. from the ZP habitat were 64.0, 82.7 and 53.3% under the 50% shade treatment, 70% shade treatment, and 90% shade treatment, respectively. Generally, the *P. fallax* Hemsl. plants from the two habitats exhibited relatively high survival under the 70% shade treatment.

### Effects of light intensity on the biomass of *Polygala fallax* Hemsl.

Table 1 shows that the biomass and the root-shoot ratio of *P. fallax* Hemsl. plants differed significantly among the shade treatments but not between locations, and significant interactions in terms of root biomass and the root-shoot ratio occurred between the shade treatments and locations. For the *P. fallax* Hemsl. plants from the LC habitat, the root biomass, aboveground biomass and total biomass were the highest under the 70% shade treatment, followed by the 50% shade treatment, and were the lowest under the 90% shade treatment. The root biomass, aboveground biomass and total biomass of *P. fallax* Hemsl. plants from the ZP habitat and the root-shoot ratio of *P. fallax* Hemsl. plants from the ZP and LC habitats were the highest under the 50% shade treatment, followed by the 70% shade treatment, and they were the lowest under the 90% shade treatment. However, no significant differences in these biomass parameters of the *P. fallax* Hemsl. plants from the ZP and LC habitats were observed between the 70% shade treatment and the 50% shade treatment.

**Table 1:** Effects of shade treatments on the biomass of *Polygala fallax* Hemsl. plants.

Location	Shade treatments (%)	Root biomass (g plant^−1^)	Above-ground biomass (g plant^−1^)	Total biomass (g plant^−1^)	Root-shoot ratio
LC	50	1.22 ± 0.55^a^	1.30 ± 0.46^a^	2.53 ± 0.93^a^	0.94 ± 0.32^a^
	70	1.54 ± 0.50^a^	1.79 ± 0.64^a^	3.33 ± 1.09^a^	0.88 ± 0.19^a^
	90	0.26 ± 0.09^c^	0.57 ± 0.18^b^	0.83 ± 0.28^b^	0.45 ± 0.07^b^
ZP	50	1.43 ± 0.87^a^	1.57 ± 0.80^a^	3.00 ± 1.61^a^	0.90 ± 0.24^a^
	70	1.00 ± 0.39^ab^	1.43 ± 0.49^a^	2.43 ± 0.84^a^	0.70 ± 0.15^ab^
	90	0.40 ± 0.19^bc^	0.60 ± 0.27^b^	1.00 ± 0.45^b^	0.67 ± 0.15^ab^
Location(L)		0.24^ns^	0.03^ns^	0.11^ns^	0.00^ns^
Shade(S)		25.0^**^	22.2^**^	25.8^**^	15.8^**^
L × S		3.35^*^	1.85^ns^	2.74^ns^	5.13^**^

*, ** and ns indicate significance at *p* < 0.05, *p* < 0.01 level and not significant, respectively. Different letters indicate significant differences among the three shade treatments for the *P. fallax* Hemsl. plants from the two habitats (LC, ZP) at the *p* < 0.05 level by a least significant difference test.

### Effects of light intensity on plant characteristics of *Polygala fallax* Hemsl.

Table 2 shows that, except for leaf width, the plant characteristics (plant height, basal diameter, root length, length and width of leaves) differed significantly among the shade treatments but not between the locations, and no significant interactions occurred between the shade treatments and locations. The *P. fallax* Hemsl. plants from the LC habitat exhibited the highest values for these characteristics under the 70% shade treatment, followed by the 50% shade treatment, and showed the lowest values under the 90% shade treatment. Similar trends in plant characteristics were observed for *P. fallax* Hemsl. plants from the ZP habitat, except for root length, which was the highest under the 50% shade treatment, followed by the 70% shade treatment. However, there were no significant differences in root length for the *P. fallax* Hemsl. plants from the ZP habitat between the two shade (70 and 50%) treatments.

**Table 2:** Effects of shade treatments on the characteristics of *Polygala fallax* Hemsl. plants.

Location	Shade treatments (%)	Plant height	Basal diameter	Root length	Leaf length	Leaf width
LC	50	21.6 ± 4.23^bc^	3.99 ± 0.73^ab^	28.6 ± 5.88^ab^	14.1 ± 1.87^b^	3.89 ± 0.37^c^
	70	26.5 ± 6.26^a^	4.34 ± 0.80^a^	31.2 ± 5.93^ab^	17.3 ± 1.26^a^	4.66 ± 0.30^ab^
	90	19.4 ± 4.28^cd^	2.98 ± 0.38^c^	26.6 ± 6.04^b^	12.3 ± 1.90^b^	3.76 ± 0.21^c^
ZP	50	23.4 ± 7.76^ab^	3.90 ± 0.95^ab^	34.8 ± 11.93^a^	12.0 ± 3.13^b^	4.33 ± 1.15^bc^
	70	24.3 ± 6.16^ab^	3.89 ± 0.74^b^	32.9 ± 7.07^ab^	17.5 ± 2.18^a^	5.05 ± 0.54^a^
	90	17.4 ± 3.60^d^	2.90 ± 0.50^c^	30.2 ± 6.60^ab^	12.3 ± 2.38^b^	4.04 ± 0.63^c^
Location(L)		0.73^ns^	2.77^ns^	3.75^ns^	1.04^ns^	5.50^*^
Shade(S)		17.8^**^	33.9^**^	1.40^ns^	30.7^**^	14.2^**^
L × S		1.70^ns^	1.03^ns^	0.47^ns^	1.42^ns^	0.09^ns^

### Effects of light intensity on the chlorophyll content of *Polygala fallax* Hemsl.

Table 3 shows that the chlorophyll content (chlorophyll a, chlorophyll b, carotenoids) differed significantly among the shade treatments but not between locations, but there was a significant interaction between shade treatment and location, except for the chlorophyll b and carotenoid contents. The chlorophyll contents in the *P. fallax* Hemsl. plants from the two habitats (LC, ZP) exhibited a response trend similar to that found under the shade treatments. Generally, the chlorophyll a, chlorophyll b, carotenoid, and total chlorophyll contents were the highest under the 90% shade treatment, followed by the 70% shade treatment, and they were lowest under the 50% shade treatment.

**Table 3:** Effects of shade treatments on the chlorophyll contents of *Polygala fallax* Hemsl. plants.

Location	Shade treatments (%)	Chlorophyll a (mg dm^−2^)	Chlorophyll b (mg dm^−2^)	Carotenoids (mg dm^−2^)	Total chlorophyll (mg dm^−2^)
LC	50	2.46 ± 0.54^de^	0.70 ± 0.17^cd^	0.66 ± 0.1^c^	3.16 ± 0.71^de^
	70	2.96 ± 0.57^cd^	0.85 ± 0.12^c^	0.70 ± 0.11^c^	3.81 ± 0.69^cd^
	90	4.06 ± 0.41^ab^	1.27 ± 0.14^ab^	0.91 ± 0.1^ab^	5.33 ± 0.55^ab^
ZP	50	1.80 ± 0.69^e^	0.54 ± 0.19^d^	0.48 ± 0.15^d^	2.34 ± 0.88^e^
	70	3.54 ± 0.83^bc^	1.11 ± 0.23^b^	0.81 ± 0.17^bc^	4.65 ± 1.06^bc^
	90	4.42 ± 0.63^a^	1.41 ± 0.21^a^	0.99 ± 0.15^a^	5.83 ± 0.83^a^
Location(L)		0.15^ns^	1.50^ns^	0.00^ns^	0.34^ns^
Shade(S)		28.6^**^	39.7^**^	20.7^**^	31.2^**^
L × S		2.82^ns^	3.41^*^	3.79^*^	2.96^ns^

### Effects of light intensity on the photosynthetic rate of *Polygala fallax* Hemsl.

**Figure 2:**
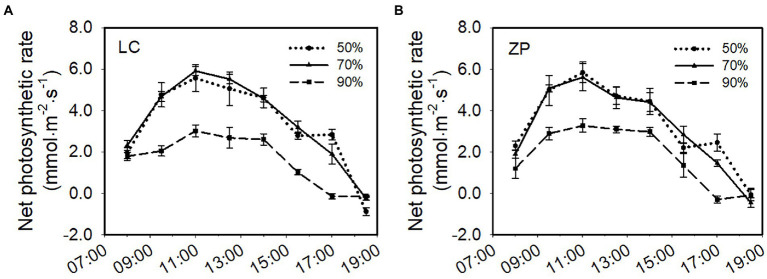
Effects of shade treatments on the net photosynthetic rate of *P. fallax* Hemsl. plants. The data are presented as the mean ± SD (*n* = 5). **(A)** Net photosynthesis rate of the *P. fallax* Hemsl. plants from the LC habitat; **(B)** Net photosynthesis rate of the *P. fallax* Hemsl. plants from the ZP habitat.

Light intensity significantly affected the photosynthetic rate of *P. fallax* Hemsl. plants. The response trends of the photosynthetic rate of *P. fallax* Hemsl. plants from the two habitats (LC, ZP) were similar under the three shade treatments. The photosynthetic rates of *P. fallax* Hemsl. plants from the two habitats were highest under the 70% shade treatment, followed by the 50% shade treatment, but no significant difference existed between the 70% shade treatment and the 50% shade treatment. The lowest photosynthetic rates were observed under the 90% shade treatment, and these values were significantly lower than the values under the 70% shade treatment and the 50% shade treatment (Figure 2).

### Effects of light intensity on the saponin concentration of *Polygala fallax* Hemsl.

**Figure 3:**
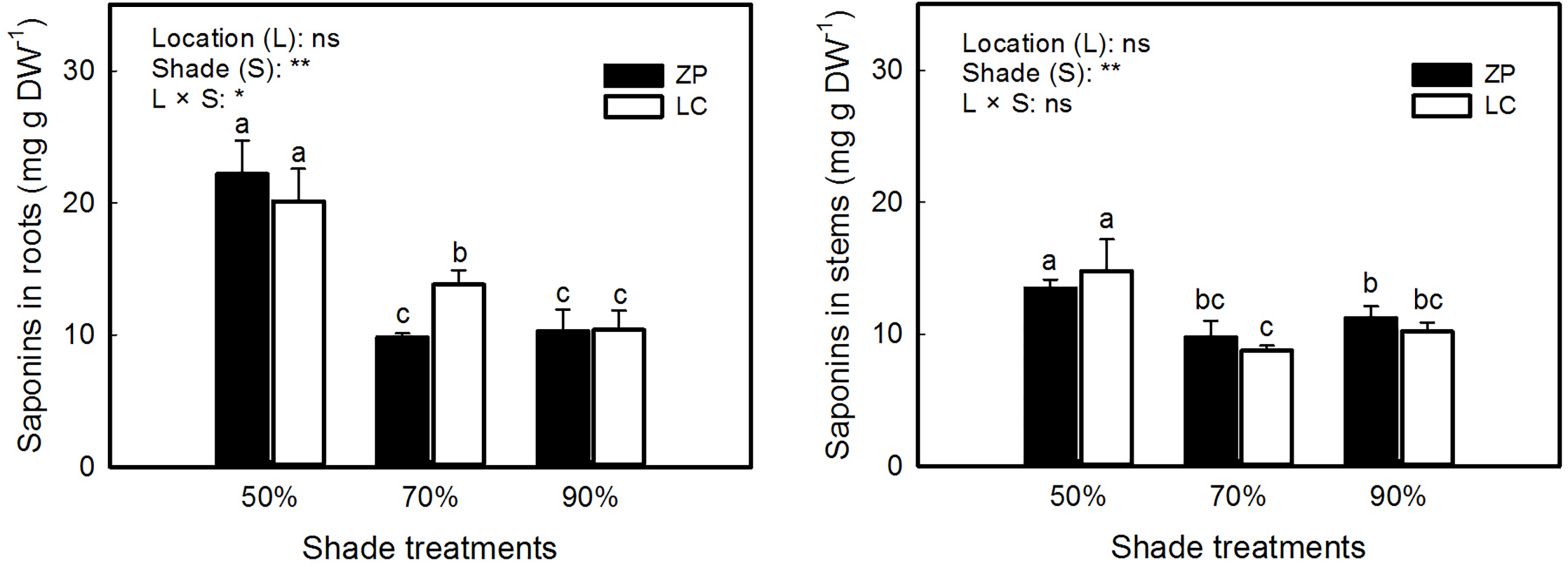
Effects of shade treatments on the saponin concentration of *P. fallax* Hemsl. plants. The data are presented as the mean ± SD (*n* = 3). *, ** and ns indicate significance at *p* < 0.05, *p* < 0.01 level and not significant, respectively. Different letters indicate significant differences among the three shade treatments for the *P. fallax* Hemsl. plants from the two habitats (LC, ZP) at the *p* < 0.05 level by a least significant difference test.

Figure 3 shows that the concentration of total saponins in *P. fallax* Hemsl. plants differed significantly among the shade treatments but not between locations, and significant interactions occurred between the shade treatments and locations in the concentration of saponins in roots but not in stems. The concentration of saponins in roots and stems of *P. fallax* Hemsl. plants from the ZP and LC habitats were significantly higher under the 50% shade treatment than under the 70 and 90% shade treatments. However, the differences in the concentration of saponins in *P. fallax* Hemsl. plants were small between the 70 and 90% shade treatments.

## Discussion

*Polygala fallax* Hemsl., a traditional Chinese medicinal plant species, is highly sensitive to irradiance. Previously, Zhang (2013) cultivated *P. fallax* Hemsl. plants under fir and broadleaf mixed forests with different canopy densities and obtained high biomass production of *P. fallax* Hemsl. under a canopy density of 0.1 ~ 0.3, followed by a canopy density of 0.3 ~ 0.5, under which the plants could survive. The lowest amounts of biomass were produced under a canopy density of 0.7 ~ 0.9. *P. fallax* Hemsl. plants seemingly grew better under weaker light conditions. Thus, we believe that *P. fallax* Hemsl. is a typical shade loving plant. However, the optimum light intensity for ensuring the best growth of *P. fallax* Hemsl. plants remains unclear.

In the current study, *P. fallax* Hemsl. plants from different habitats presented high survival rates under the 50 and 70% shade treatments (PAR of 662 μmol m^−2^ s^−1^ and 401 μmol m^−2^ s^−1^, respectively; Figure 1), which is in accordance with previous results (Zhang, 2013). We also observed that *P. fallax* Hemsl. plants exhibited the highest values for biomass and plant characteristics under the 50 and 70% shade treatments (Tables 1, 2), which can be explained by the relatively high photosynthesis rate of the leaves under these treatments (Figure 2). Thus, the *P. fallax* Hemsl. plants preferentially grew under PARs of 662 μmol m^−2^ s^−1^ and 401 μmol m^−2^ s^−1^ resulting from the 50 and 70% shade treatments. In summary, a PAR of 401 μmol m^−2^ s^−1^ – 662 μmol m^−2^ s^−1^ ensured a high leaf photosynthesis rate, resulting in high biomass production, and was associated with a relatively high survival rate. Thus, this level of irradiance is optimal for the cultivation of *P. fallax* Hemsl. plants.

Irradiance is necessary for the survival, growth, and reproduction of higher plants during their lifespan (Franklin and Forster, 1997). Excessive or insufficient light intensity can be harmful to the photosynthetic system of medicinal plants. In the present study, *P. fallax* Hemsl. plants exhibited a higher photosynthesis rate under the 50% shade treatment and 70% shade treatment but a lower photosynthesis rate under the 90% shade treatment (Figure 2). High light stress reduced photosynthesis due to (i) the blocked electron transport during photosynthesis and (ii) the irreversible inactivation of the photosystem II (PSII) reaction center (Lu et al., 2017), while weak light limited photosynthesis due to the resulting dark-induced senescence (Spundova et al., 2005; Parlitz et al., 2011). We speculated that full light may induce high light stress injury to the photosynthetic system and that the serious shading (PAR of 131 μmol m^−2^ s^−1^) resulting from the 90% shade treatment induced weak light stress injury to the *P. fallax* Hemsl. plants.

Additionally, we observed that the chlorophyll content decreased in *P. fallax* Hemsl. plants from both the LC and ZP habitats as the light intensity increased (Table 3). Shade treatment was shown to reduce the chlorophyll content in soybean, rice, cotton, American grapevine and Chinese tea plants (Tian et al., 2017; Li et al., 2019; Silva et al., 2019; Hussain et al., 2020; Zhang et al., 2020). Moreover, the chlorophyll contents of *Camellia japonica* ‘Naidong’ and maize plants decreased under shade treatment, as was observed in other plant species (Ren et al., 2016; Liu et al., 2018). Xie et al. (2020) reported that chlorophyll a and chlorophyll b contents decreased in C_3_ turfgrasses but increased in C_4_ turfgrasses under shaded conditions. The responses of chlorophyll to light intensity seemingly vary among species. *P. fallax* Hemsl. plants prefer weaker light intensity and thus may accumulate more chlorophyll under shaded conditions. In tea plants, chlorophyll and carotenoid synthesis-related genes were downregulated in response to strong light but were upregulated in response to shade or reduced light intensity (Yue et al., 2021). Genes involved in the biosynthesis of chlorophyll in *P. fallax* Hemsl. plants under shade treatment should be further studied.

As one of the most important active ingredients in *P. fallax* Hemsl. plants, saponins were the highest under the 50% shade treatment (Figure 3). In *Panax japonicus* var. *major*, the total saponins were enhanced by shade treatment when compared with nonshade treatment (Huang et al., 2018). However, the total saponins in the roots and stems of *P. fallax* Hemsl. plants decreased as shade increased (Figure 3), which may be due to the expression of key enzymes involved in saponin biosynthesis being downregulated by more serious shading (Cheng et al., 2021). Thus, an optimum light intensity is crucial for saponin biosynthesis in this medicinal plant. In the present study, we found that a PAR of 662 μmol m^−2^ s^−1^ resulting from the 50% shade treatment contributed to the relatively high accumulation of saponins in *P. fallax* Hemsl. plants.

## Conclusion

The *P. fallax* Hemsl. plants exhibited a high leaf photosynthesis rate, produced high biomass and presented a relatively high survival rate under a PAR of approximately 401 μmol m^−2^ s^−1^ – 662 μmol m^−2^ s^−1^. The greatest concentration of total saponins in *P. fallax* Hemsl. plants were achieved under a PAR of 662 μmol m^−2^ s^−1^. In conclusion, a PAR of approximately 662 μmol m^−2^ s^−1^ should be adopted for the cultivation of *P. fallax* Hemsl. plants for obtaining both high biomass production and a high yield of saponins.

## Data availability statement

The original contributions presented in the study are included in the article/supplementary material, further inquiries can be directed to the corresponding author.

## Author contributions

HL, MW, and HT designed experiments. HL, XH, LW, XZ, and BL performed experiments. CW and BL analyzed data and compiled figures. CW wrote the manuscript. CW and HT edited the final manuscript. All authors contributed to the article and approved the submitted version.

## Funding

This work was supported by the Science and Technology Major Project of Guangxi, China (grant no. Guike AA22096020), the Guangxi Natural Science Foundation (grant no. 2021GXNSFBA220067), the Guangxi Key Laboratory of Functional Photochemical Research and Utilization lab’s projects (grant no. 20190213-2), and the Basic Research Fund of Guangxi Academy of Sciences (grant no. CQZ-C-1901).

## Conflict of interest

The authors declare that the research was conducted in the absence of any commercial or financial relationships that could be construed as a potential conflict of interest.

## Publisher’s note

All claims expressed in this article are solely those of the authors and do not necessarily represent those of their affiliated organizations, or those of the publisher, the editors and the reviewers. Any product that may be evaluated in this article, or claim that may be made by its manufacturer, is not guaranteed or endorsed by the publisher.
